# Navigation Error Characteristics of LIO-, VIO-, and RIMU-Assisted INS/GNSS Multi-Sensor Fusion Schemes in a GNSS-Denied Environment

**DOI:** 10.3390/s26072068

**Published:** 2026-03-26

**Authors:** Kai-Wei Chiang, Syun Tsai, Chi-Hsin Huang, Yang-En Lu, Surachet Srinara, Meng-Lun Tsai, Naser El-Sheimy, Mengchi Ai

**Affiliations:** 1 Department of Geomatics, National Cheng Kung University, Tainan City 701, Taiwan; kwchiang@geomatics.ncku.edu.tw (K.-W.C.);; 2 Department of Civil Engineering, University of Phayao, Phayao 56000, Thailand; 3 High-Definition Maps Research Center, Department of Geomatics, National Cheng Kung University, Tainan City 701, Taiwan; 4 Department of Geomatics Engineering, University of Calgary, Calgary, AB T2N 1N4, Canada

**Keywords:** integrated navigation system, inertial measurement unit (IMU), visual inertial odometry (VIO), LiDAR inertial odometry (LIO), redundant inertial measurement unit (RIMU), boresight angle calibration

## Abstract

Autonomous vehicles at level 3 and above must maintain high navigation accuracy, particularly in global navigation satellite system (GNSS)-denied environments. The main innovations of this work are threefold. First, we integrate visual inertial odometry (VIO) and light detection and ranging (LiDAR) inertial odometry (LIO) as external updates to mitigate the rapid drift of micro-electromechanical system (MEMS)-based industrial-grade inertial measurement units (IMUs) during long-term GNSS outages. Second, we adopt a redundant IMU (RIMU) approach that fuses multiple low-cost IMUs to reduce sensor noise and improve reliability. Third, we propose a system calibration methodology using both static and dynamic vehicle motion to estimate extrinsic parameters (boresight angles and lever arms) of the sensors, achieving an overall boresight angle root-mean-square error of 0.04 degrees in the simulation. Experiments were conducted under a 7 min GNSS-denied scenario in an underground parking lot, allowing for comparison of the error characteristics of multi-sensor fusion schemes against a navigation-grade reference. The INS/GNSS/LIO framework achieved a two-dimensional root-mean-square position error of 1.22 m (95% position error within 2.5 m), meeting the lane-level (1.5 m) accuracy requirement under a GNSS outage exceeding 7 min without prior maps. In contrast, the RINS/GNSS/VIO framework yielded a 4.71 m 2D mean position error under the same conditions. This paper provides a quantitative comparison of the baseline error characteristics of VIO-, LIO-, and RIMU-assisted INS/GNSS fusion under a GNSS-denied navigation scenario.

## 1. Introduction

Road vehicle autonomy is classified by the Society of Automotive Engineers into six levels, with conditional automation (level 3) denoting the threshold at which a vehicle is considered autonomous. For level 3 or higher, the vehicle system is required to independently monitor the driving environment and perform dynamic driving tasks [1], which require high positioning availability and accuracy to ensure safety in navigation. The positioning requirements of autonomous vehicles can be categorized into three levels—namely, road (5 m), which lane (1.5 m), and where-in-lane (0.5 m)—with an additional active control level (0.1 m) [2]. To achieve level 3+ autonomy, vehicle positioning requires at least which-lane (1.5 m) accuracy.

Vehicle navigation applications generally integrate a global navigation satellite system (GNSS) with an inertial navigation system (INS). However, these applications often involve partial or complete loss of GNSS signals. A land vehicle with an INS/GNSS can achieve positioning accuracy of up to <0.4 m under full satellite visibility, corresponding to “which lane” accuracy (1.5 m) [3]. However, such a vehicle may experience considerable positioning accuracy degradation in a GNSS-denied environment, such as dense urban environments [4,5], or when it receives non-line-of-sight signals [6]. When a vehicle enters an environment with limited GNSS coverage, its INS/GNSS functionality effectively degrades to that of a pure INS [7]. In this scenario, inertial measurement unit (IMU) errors (e.g., bias and scale factor errors) may amplify positioning errors [8]. INS drift, which is influenced by IMU quality, strongly affects the performance of navigation systems over time. While high-grade IMUs mitigate INS drift, they are cost-prohibitive for widespread commercialization. Consequently, current land vehicle navigation systems typically rely on low-cost INSs and auxiliary sensors (e.g., odometers, cameras, and light detection and ranging [LiDAR] systems) [4,9,10].

The use of a low-cost IMU in an INS typically leads to poor bias stability, causing considerable drift under pure inertial conditions. In this context, the integration of multiple IMUs may reduce noise and increase positioning reliability [11,12]. Researchers have also attempted to mitigate INS drift by combining odometry algorithms, such as visual odometry and LiDAR odometry, the errors of which increase with distance traveled rather than elapsed time. For visual odometry, online bias estimation and related techniques can be applied [13,14]. While uncompensated IMU bias can cause the accumulation of pose estimation errors [15], inertial measurements can help to recover the scale for monocular visual odometry. Online bias estimation can also be applied to LiDAR odometry. LiDAR provides consistent range measurements and dense point clouds, offering reliable motion constraints for an IMU through point cloud matching algorithms [16,17]. The IMU de-skews point clouds by providing high-frequency motion information under highly dynamic conditions [18]. Loop closure or map-matching techniques can improve both visual and LiDAR odometry strategies; however, these algorithms are effective only within a known environment. To evaluate the worst-case scenario—namely, the characteristic baseline error without the influence of external corrections for different fusion strategies—and focus on examining error behaviors in relation to drift, we do not apply loop closure or map matching in this study.

This research focuses on three cost-effective strategies involving micro-electromechanical system (MEMS)-based industrial-grade IMUs and low-cost sensors. In particular, a redundant IMU (RIMU) approach for reducing noise and increasing positioning reliability is combined with visual inertial odometry (VIO) and LiDAR inertial odometry (LIO), which facilitates external measurement within an INS/GNSS. Given that GNSS-denied scenarios are generally characterized by rich environmental features, this integrated system exploits environmental features for robust odometry. We analyzed the error characteristics of VIO-, LIO-, and INS-based approaches to compare their performance and cost-effectiveness in GNSS-denied environments.

The main contributions of this study are as follows:

- The cost-effectiveness of VIO-, LIO-, and RIMU-based INS/GNSS multisensor fusion schemes is compared.
- A novel methodology for system calibration involving static and dynamic vehicle motions is developed to estimate the external parameters of systems installed in a vehicle.
- A long-term GNSS outage scenario (up to 7 min) is tested to analyze the error characteristics of VIO, LIO, and INSs.

## 2. Methodology

This section introduces the proposed multisensor fusion scheme for INS/GNSS deployment, including the filter structure, motion constraints, velocity update, and heading change update through inertial-aided odometry. It also describes the advantages of inertial-aided odometry over visual odometry or LiDAR odometry, and summarizes the selected VIO (VINS-Fusion) and LIO (FAST-LIO 2.0) algorithms. Finally, it details the theoretical principles and cost-effectiveness of the RIMU application and outlines the adopted system calibration procedures.

### 2.1. Multisensor Fusion Scheme

**Figure 1:**
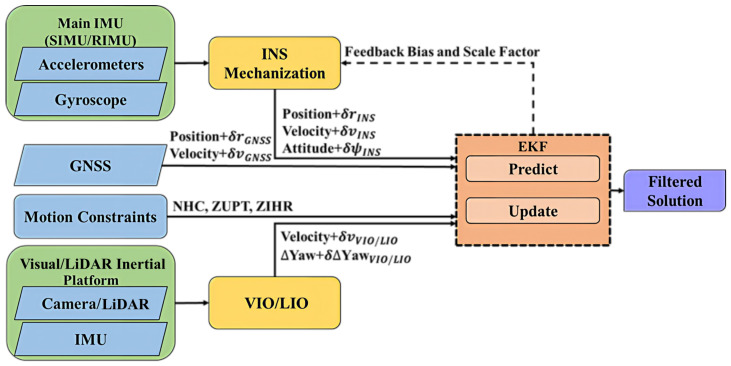
Flowchart of the proposed multisensor fusion scheme.

INS/GNSS frameworks are the most widely applied architecture in modern navigation systems [10,19]. An INS is typically adopted as the primary system in the navigation filter of such frameworks, due to its high sampling frequency and independence from the environment. However, INSs are subject to error accumulation if IMU sensor errors are not properly compensated, such as bias and scale factor errors. In particular, gyroscope bias error leads to attitude error accumulation, subsequently causing the gravity vector to be projected onto the wrong accelerometer axis and resulting in the accumulation of velocity and position errors. To estimate these error sources, an INS and a GNSS are typically integrated with an extended Kalman filter (EKF). In the present study, a loosely coupled integration structure is applied. The displacements obtained from the INS and GNSS can be compared to determine the bias and scale factor errors, and the error sources (e.g., bias, scale factor, and GNSS) can be compensated. After the sources of INS error are correctly estimated, error accumulation can be confined within acceptable limits. INS error estimation is thus the primary challenge when aiming to establish a stable navigation system. External measurements from secondary sensors or virtual measurements, such as motion constraints, can improve the performance of a navigation filter. Therefore, we developed the multisensor fusion scheme illustrated in Figure 1.

#### 2.1.1. Loosely Coupled INS/GNSS Integration

INS navigation equations are typically nonlinear; thus, it is necessary to linearize the system and measurement models to satisfy the assumptions for the application of Kalman filters. The INS/GNSS scheme applied in this study follows that outlined in [20]. The state vector of the EKF is defined as follows:

(1) $$x_{k}={\left[r\;v\;\phi \;b_{a}\;b_{g}\;S_{a}\;S_{g}\right]}_{21\times 1}^{T}$$

(2) $${\hat{x}}_{k}=x_{k}+\delta x_{k}$$

where *x* is the state vector, which contains the position *r*, velocity *v*, attitude ϕ, accelerometer bias $b_{a}$, gyroscope bias $b_{g}$, accelerometer scale factor $S_{a}$, and gyroscope scale factor $S_{g}$; δx is the error state vector; and ${\hat{x}}_{k}$ is the updated state at time *k*.

The EKF system and measurement models are fundamental for state prediction and updating, which are implemented in discrete-time form as follows:

(3) $$\delta x_{k+1}=\Phi_{k,k+1}\delta x_{k}+w_{k}$$

(4) $$\delta z_{k}=H_{k}\delta x_{k}+e_{k}$$

where $\Phi_{k,k+1}$ represents the state transition matrix, $w_{k}$ denotes the white noise sequence of the system, $H_{k}$ is the design matrix for the projection of states into the measurement space, and $e_{k}$ indicates the white noise sequence of these measurements.

For land vehicle applications, motion constraints are commonly applied to improve the performance. In this study, we applied zero-velocity update (ZUPT) and zero-integrated heading rate (ZIHR) when the vehicle is in static motion. A non-holonomic constraint (NHC) is also applied, and we assumed that the vehicle does not experience side slip and moving on a plane.

#### 2.1.2. Velocity Update

Figure 1 displays the proposed multisensor fusion scheme with velocity update provided by inertial-aided odometry (i.e., VIO and LIO).

The velocity of the odometry platform is described in the odometry platform (*p*) frame $v_{np}^{p}$, which represents the body frame of each built-in IMU. The velocity estimated using the EKF is the velocity of the main IMU body (*b*) in the navigation (*n*) frame $v_{nb}^{n}$. The measurement can be updated according to the relationship between $v_{np}^{p}$ and $v_{nb}^{n}$. This relationship follows rigid body dynamics, as indicated in the following equation:

(5) $$v_{np}^{p}=R_{b}^{p}R_{n}^{b}v_{nb}^{n}+R_{b}^{p}\left(\omega_{nb}^{b}\times r_{bp}^{b}\right)$$

where $R_{n}^{b}$ is the rotation matrix from frame *n* to frame *b* of the main IMU body, $R_{b}^{p}$ is the rotation matrix from frame *b* to frame *p*, $\omega_{nb}^{b}$ is the angular rate of the main IMU body in frame *b*, and $r_{bp}^{b}$ is the lever arm from the main IMU body to the odometry platform in frame *b*. The parameters $R_{n}^{b}$, $v_{nb}^{n}$, and $\omega_{nb}^{b}$ are estimated using the EKF. The parameters $R_{b}^{p}$ (rotation matrix of the boresight angle) and $r_{bp}^{b}$ are pre-estimated using the system calibration procedure introduced in Section 2.5. Rather than the velocity of the odometry platform in frame *n*, this method uses the velocity in frame *p* to avoid introducing accumulated heading drift. The measurement update process is analogous to the application of a wheel odometer; however, the measured velocity is a three-dimensional (3D) vector. Furthermore, VIO and LIO incorporate heading change measurements to control the INS heading error. Further details regarding the heading change update are provided in the following section.

#### 2.1.3. Heading Change Update

To account for the heading drift with VIO and LIO, the proposed integrated scheme adopts a heading change update instead of a heading update. As the attitude estimation steps in VIO and LIO involve the use of accelerometers to achieve leveling, the attitude reference frame is in the same plane as frame *n*, with the first frame regarded as the heading reference instead of true north. In this case, the heading change can be projected to frame *n* by correcting the heading. After projecting the heading change to frame *n*, the heading change of the odometry platform in frame *n* is obtained as ${\dot{\varphi }}_{p}^{n}$. The attitudes of the odometry platform ($R_{n}^{p}$) and main IMU body ($R_{n}^{b}$) are aligned through the boresight rotation $R_{b}^{p}$. The heading, pitch, and roll angle of $R_{n}^{b}$ are denoted as φ, θ, and ϕ, respectively. The heading change is influenced by the EKF state and gyro bias $b_{g}$, as indicated in Equation (7):

(6) $$R_{n}^{p}=R_{b}^{p}R_{n}^{b}$$

(7) $$\delta z_{\dot{\varphi }}={\hat{\dot{\varphi }}}_{b}^{n}-{\tilde{\dot{\varphi }}}_{p}^{n}=\left[0\;sec\theta sin\phi \;sec\theta cos\phi \right]\left(b_{g}+n\right)-e_{\dot{\varphi }}$$

where $\delta z_{\dot{\varphi }}$ is the error of the heading change measurement; ${\hat{\dot{\varphi }}}_{b}^{n}$ is the predicted heading change; ${\tilde{\dot{\varphi }}}_{p}^{n}$ is the measured heading change (from VIO or LIO); $b_{g}$ is the gyro bias; *n* is the noise; and $e_{\dot{\varphi }}$ is the measurement noise.

### 2.2. VIO Algorithm (VINS-Fusion)

The VINS-Fusion algorithm integrates a camera with a low-cost IMU to estimate the six-degrees-of-freedom (DOFs) navigation state. In contrast to vision-only systems, such as PTAM [21], SVO [22], LSD-SLAM [23], DSO [24], and ORB-SLAM [25], the IMU-based measurement of VIO can be used to estimate scale parameters, recover the scale of the world, observe the roll and pitch angles, and pad the gap between track losses. An IMU is generally considered a complementary sensor for vision-based algorithms, as its sampling rate is considerably higher than that of a camera, enabling it to track high-speed motion. Vision-based algorithms can also compensate for IMU drift. We selected VINS-Mono [13], a tightly coupled nonlinear-optimization-based algorithm, to improve our INS/GNSS architecture. In this approach, visual feature point observations and pre-integrated IMU measurements are used to estimate the state of motion, and the extrinsic association between the camera and IMU, as well as the IMU bias, can be calibrated online. Moreover, optical flow algorithms—which track feature points across image sequences—lead to faster computation time when compared with the descriptor-based methods, and are thus more suitable for real-time applications.

The VINS-Fusion algorithm is an advanced version of VINS-Mono, which functions as a separate system capable of providing independent measurements. This system comprises a monochrome camera with an IMU to provide information on an absolute scale, but at low cost and with a flexible configuration, making it suitable for autonomous vehicles. The velocity and heading change provided by VINS-Fusion are adopted to update the INS/GNSS system.

### 2.3. LIO Algorithm (FAST-LIO 2.0)

FAST-LIO employs a tightly coupled iterated EKF to fuse LiDAR feature points with IMU data [26,27]. LiDAR-only odometry approaches—including the iterative closest point method [28], LOAM [29] and its variant LeGO-LOAM [30], and LOAM-Livox [31]—tend to degenerate in feature-poor environments, whereas the inclusion of IMU measurements helps to mitigate this effect. Tightly coupled LiDAR–inertial methods, such as LINS [32] and LIPS [33], fuse raw feature points directly with IMU measurements; this differs from loosely coupled schemes such as IMU-aided LOAM [34], LION [35], and those described in [36,37].

FAST-LIO 2.0 is an advanced framework for improving LiDAR navigation and mapping based on FAST-LIO. This method involves the direct registration of raw LiDAR points to the map, eliminating the need for feature extraction and simplifying navigation in challenging environments, such as areas with small fields of view or structureless areas. Moreover, FAST-LIO2 employs a novel data structure—namely, the ikd-tree (incremental kd-tree)—to support real-time mapping through efficient map updates.

Similar to VINS-Fusion, FAST-LIO 2.0 operates as a separate system to provide independent measurements. The velocity and heading change provided by FAST-LIO 2.0 are adopted to update the INS/GNSS.

### 2.4. Redundant IMU

The basic concept of an RIMU is derived through error propagation. If the observations of *N* IMUs with the same specifications and axial direction are averaged, the noise σ can be reduced by a factor of $1/\sqrt{N}$, considerably decreasing the gyro bias instability. Under the same noise magnitude for all IMUs, such as that described in Equation (8), when the *i*-th IMU provides measurement $X_{i}$ with noise $\sigma_{X_{i}}$, the propagated error can be derived using Equation (9). Redundant observations can prevent failures caused by a single defective IMU or any erroneous data, increasing system reliability.

(8) $$∵\bar{X}=\frac{\sum_{i=1}^{N}X_{i}}{N},\;\sigma_{X_{i}}=\sigma \;$$

(9) $$∴\sigma_{\hat{X}}=\sqrt{\sum_{i=1}^{N}\frac{\sigma_{X_{i}}^{2}}{N^{2}}}=\sqrt{N\cdot \frac{\sigma^{2}}{N^{2}}}=\frac{\sigma }{\sqrt{N}}$$

The performance enhancement of INS/GNSS integration algorithms for a consumer-grade MEMS-based RIMU in land vehicles was analyzed to verify the competitiveness benefits provided by a tactical-grade RIMU. The Allan deviation of gyros (*z*-axis) exhibits a decrease in bias instability when passing from a single IMU to an RIMU. Numerous researchers have investigated the optimal geometry [38,39,40], systematic error reduction [41,42], and lever arm effect [43] for RIMUs, and various algorithms have been employed for data fusion and fault detection [40,44]. A recent work has demonstrated that fusion strategies for MEMS redundant-IMU arrays can achieve near-tactical-grade performance [12]. However, the present study focused only on the effectiveness of noise reduction, evaluated using Equation (9). Given that MEMS technology reduces IMU volume, power consumption, and cost, multiple IMUs can be combined in a single printed circuit board. The proposed system integrates three IMUs (ASM330LHH) with aligned axes and has a negligible lever arm. These three IMUs are represented as a single virtual IMU in our INS/GNSS algorithms. Theoretically, the gyro bias instability of the virtual IMU should decrease to $1/\sqrt{3}$ times that of a standard IMU. This principle was tested, and the results are presented in Table 1. The RIMU not only considerably decreased gyro bias instability, but it is also cheaper than a tactical-grade IMU. We conducted experimental testing under a GNSS outage of up to 7 min to evaluate the degree to which an RIMU improves drift reduction in environments characterized by extended GNSS denial. We also adopted a reliable reference system with a navigation-grade IMU to analyze the accuracy of the position, velocity, and attitude solutions obtained with the developed INS/GNSS framework.

### 2.5. System Calibration

**Figure 2:**
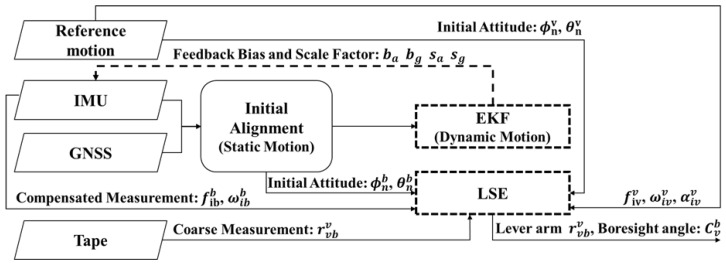
Flowchart of system calibration process.

The system calibration of individual sensors, during which the external parameters of all systems are estimated, is a prerequisite step in the proposed multisensor fusion scheme. The boresight angle and lever arm between pairs of sensors or systems must be determined to transform measurements into a standard frame, and the lever arm between the IMU and the GNSS antenna must be determined to achieve sensor fusion. Moreover, the determination of the lever arm and boresight angle between the IMU and the rear wheel allows for the application of a nonholonomic constraint for system enhancement. A vehicle frame (*v*) centered at the rear wheel was thus established as a common reference for sensor integration, and an experiment was conducted by employing a navigation-grade IMU and a GNSS to generate a reliable reference motion (this process is detailed in the following section). The reference motion is transformed into the vehicle frame *v* for estimation of the external parameters using commercial INS/GNSS integration software (Inertial Explorer 10.00, Waypoint/NovAtel Inc., Calgary, AB, Canada). System calibration is then conducted through a two-stage offline and online procedure. All sensors are assumed to be fixed on the vehicle after mounting. In the offline stage (Figure 2), external parameters between the IMU and the vehicle are estimated through simultaneous data collection with a reference system, with all systems encompassing static and dynamic vehicle motions. The lever arms of the GNSS and vehicle are coarsely measured, and the initial boresight angle (derived from the initial attitude) and the bias and scale factor of the IMU are estimated using a simple EKF for an INS/GNSS framework. Compensated measurements and reference motions are input into a least squares estimator to determine the external parameters of the IMU on the basis of rigid body dynamics equations, which are presented as Equations (10) and (11). The vehicle’s accurate motion, including its acceleration $f_{iv}^{v}$ and angular rate $\omega_{iv}^{v}$, is complemented by a trustable reference that provides the angular acceleration $\alpha_{iv}^{v}$. After compensation, the measurements $f_{ib}^{b}$ and $\omega_{ib}^{b}$ contain only white noise.

Finally, to resolve the six unknowns, the least-squares estimator is applied to dynamic data, typically collected while driving the vehicle along a figure-eight path for several turns. Through this procedure, any system with an IMU can be calibrated while it is mounted on the vehicle. All systems can be transformed into the standard frame *v*, ensuring effective measurement fusion. Given that VIO and LIO systems are typically commercial modules that integrate sensors (camera or LiDAR) with IMUs and mature algorithms, parameter calibration between the sensor and built-in IMU is usually performed in the factory. This type of system is highly flexible and thus suitable for multiple applications. The calibration procedure can be adopted to transform odometry solutions into the frame *v*, facilitating measurement fusion and the analysis of each system.

(10) $$\omega_{ib}^{b}=R_{v}^{b}\omega_{ib}^{b}\;$$

(11) $$f_{ib}^{b}=R_{v}^{b}f_{ib}^{b}+R_{v}^{b}\left(\alpha_{iv}^{v}\times r_{vb}^{v}\right)+\;R_{v}^{b}\left(\omega_{iv}^{v}\times \left(\omega_{iv}^{v}\times r_{vb}^{v}\right)\right)$$

## 3. Experiments

In this study, we analyze the error behaviors (estimated position drift) of INS/GNSS, VIO, and LIO frameworks, and the changes in these errors after integrating multiple sensors to create INS/GNSS/VIO, INS/GNSS/LIO, and RIMU-based INS (RINS)/GNSS frameworks. To evaluate the performance of the proposed calibration method, we performed a simulation with the system calibration algorithm, obtaining the external parameters of different multisensor fusion systems and acquiring a consistent baseline for system comparison. In the experiments, a high-end INS/GNSS integrated navigation framework was carried onboard a vehicle as a reference system to obtain ground-truth information. Given that both the reference system and the INS/GNSS navigation framework required GNSS convergence, each experiment began in an open-sky environment with high GNSS availability before moving to a long-distance, GNSS-denied environment to simultaneously evaluate error behavior across all systems.

### 3.1. Calibration Simulation

We verified the external parameter system calibration method described in Section 2.5 through IMU simulation. Critical IMU parameters, including bias instability, bias repeatability, and random walk for both the accelerometer and gyroscope, were set to be identical to those of the IMUs used in the experiments. In the simulation, three boresight angles were randomly altered across 100 simulations within the range of −30° to 30°. The IMU measurement was simulated according to a reference trajectory based on the desired IMU parameters and boresight angle. The results were then used to evaluate whether the performance of the calibration method was suitable for our application. In the simulation, the trajectory began from the start of the experiment route, including 400 s of static motion from the beginning of the route and 200 s of dynamic motion in an “inverted-eight” (∞) shape.

### 3.2. Vehicle Configuration Description

**Figure 3:**
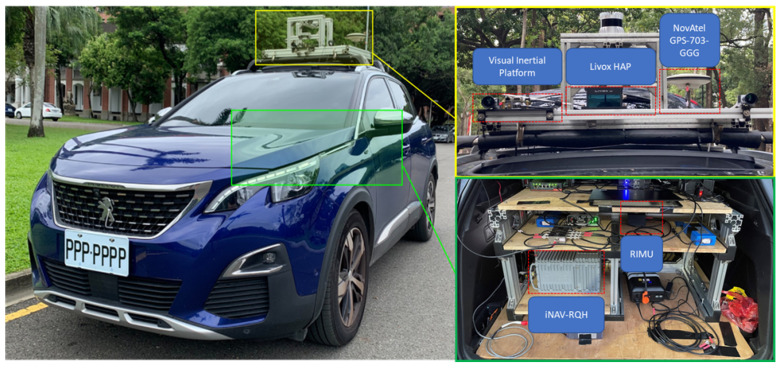
Configuration of the navigation sensors on the experimental car.

A navigation-grade IMU (iNAV-RQH; iMAR Navigation GmbH, St. Ingbert, Germany) and a GNSS receiver (NovAtel OEM7; NovAtel Inc., Calgary, AB, Canada) were installed on a land vehicle to evaluate the calibration accuracy. The Inertial Explorer 10.00 (Waypoint/NovAtel Inc., Calgary, AB, Canada) INS/GNSS integration software was used to generate a reliable reference trajectory during post-processing on the basis of IMU data from the iNAV-RQH IMU and GNSS data from the NovAtel OEM7 receiver. (The navigation-grade fusion trajectory still experienced drift during GNSS outage; comparing the forward and backward filtered trajectory position misclosure between the INS and GNSS at the end of the outage, the combined smoothed reference trajectory’s position error was about 0.2 to 0.5 m in the horizontal direction and about 1 m in the vertical direction.) Table 2 presents the specifications of the iNAV-RQH IMU. The test system included a module containing three RIMUs (ASM330LHH; STMicroelectronics N.V., Geneva, Switzerland) and a self-assembled visual platform (consisting of a low-cost IMU and an industrial camera). The industrial camera for the VIO system was a Basler acA1300-75gc paired with an 8 mm ICL-DM0824I-5M C-mount lens, both sourced from Basler AG (Ahrensburg, Germany). The camera and lens specifications for the VIO system are provided in Table 3, and the specifications of the solid-state Livox HAP LiDAR (Livox Tech Co., Ltd., Shenzhen, China) for the LIO system are provided in Table 4. To control for relevant variables, all systems used the same GNSS source with full constellations, dual frequencies, and differential GNSS, employing the NovAtel GPS-703-GGG antenna (NovAtel Inc., Calgary, AB, Canada). The configuration of the navigation sensors on the experimental vehicle is displayed in Figure 3.

### 3.3. Environment Description

**Figure 4:**
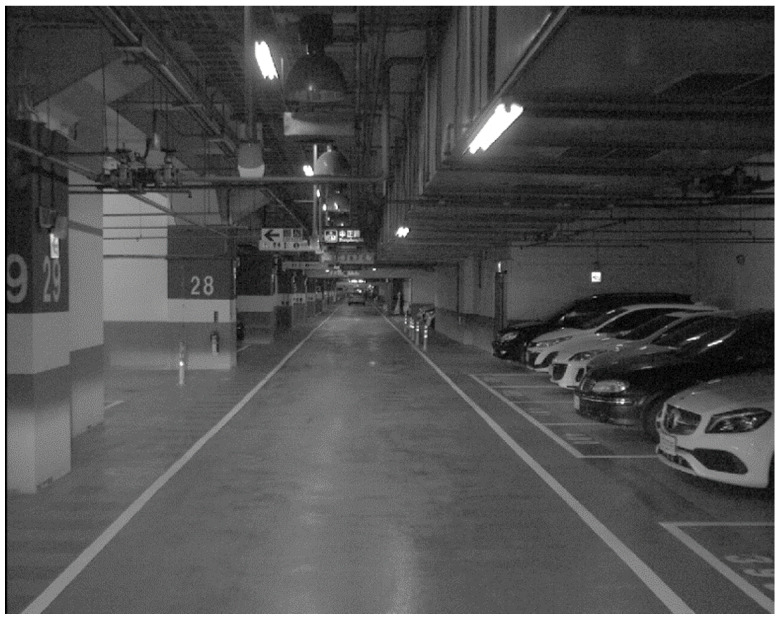
Image of the experiment field (Basler acA1300-75gc).

**Figure 5:**
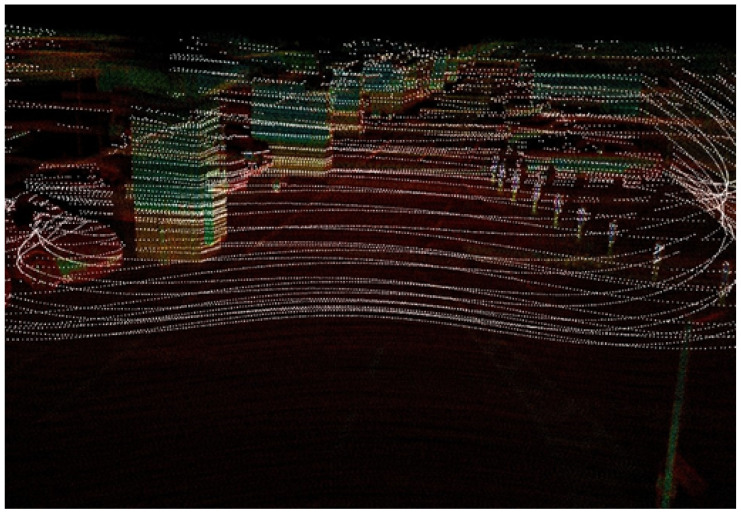
Point cloud of the experiment field (Livox HAP).

The experiments were conducted in a long, straight underground parking lot with a total length of approximately 630 m. As illustrated in Figure 4 and Figure 5, the parking lot has features for applying both visual and LiDAR odometry. The car entered the parking lot from the right-hand side of Area A, traveled to the end of the parking lot (Area D), and returned after making a U-turn, resulting in a total experimental trajectory of approximately 1260 m. The total time spent in the underground parking lot was approximately 420 s.

## 4. Results and Discussion

This section is separated into three parts. Section 4.1 presents the simulation results regarding the calibration of the boresight angle system. Section 4.2 provides a performance comparison for the VIO, LIO, and INS/GNSS frameworks. Finally, Section 4.3 details the performance of four fusion systems; namely, INS/GNSS/LIO, INS/GNSS/VIO, RINS/GNSS, and RINS/GNSS/VIO. The positioning error of an INS increases over time, whereas that of visual or LiDAR odometry increases with travel distance. However, the error-drift characteristics of VIO and LIO systems have not been previously analyzed. Therefore, we also analyzed the effects of time and travel distance on the positioning errors of these systems.

### 4.1. Simulation Result for System Calibration

**Figure 6:**
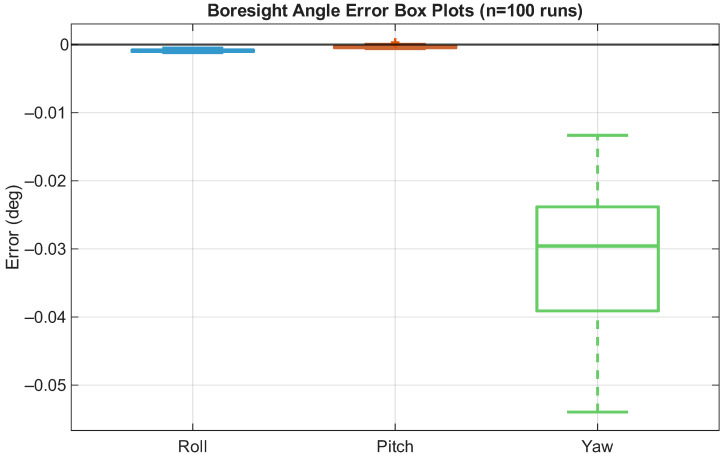
Calibration simulation results.

This section details the results of the boresight angle system calibration simulation. Figure 6 presents the boresight angle system calibration simulation results, in which the box plot illustrates the distribution of the estimated boresight angle error. The statistical results are provided in Table 5, from which it can be seen that the largest error occurred in the yaw angle. As land vehicles primarily rotate along the yaw axis, measurements along this axis tend to exhibit relatively large variations. Although the yaw angle error was the largest, the maximum error was 0.0692°, which is sufficiently small for our experiment field.

### 4.2. Performance Evaluation of the INS/GNSS, LIO, and VIO Frameworks

**Figure 7:**
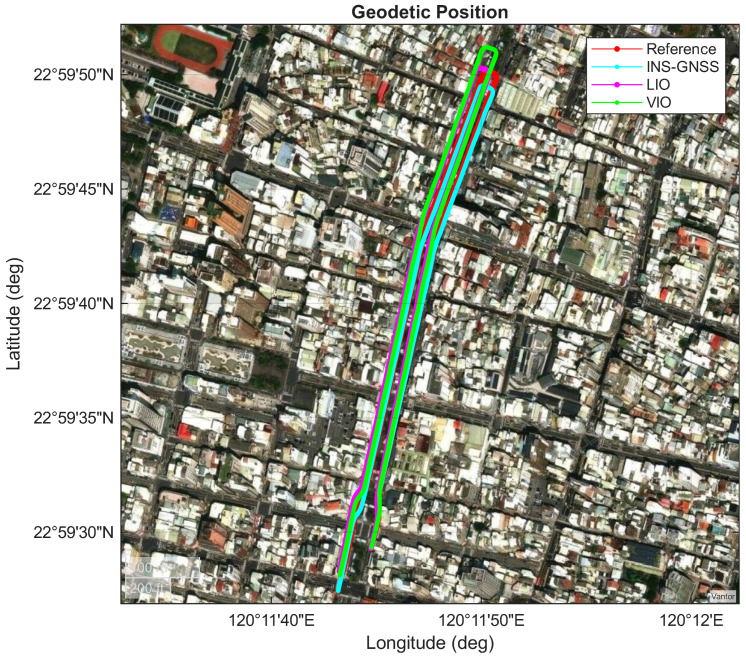
Overview of the performances of the adopted INS/GNSS, LIO, and VIO frameworks.

**Figure 8:**
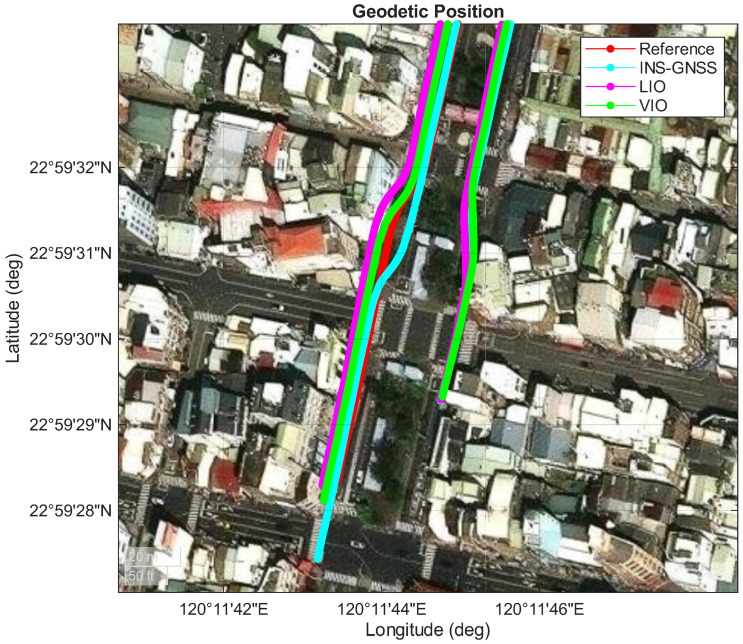
Performances of the adopted INS/GNSS, LIO, and VIO systems in Area A.

**Figure 9:**
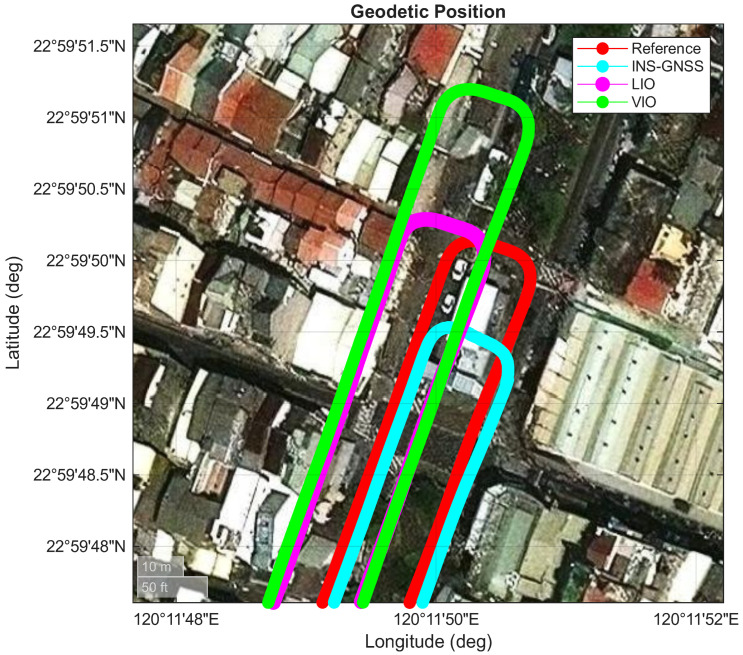
Performances of the adopted INS/GNSS, LIO, and VIO systems in Area D.

This section presents a comparison of the experimental performance of three systems—namely, INS/GNSS, LIO, and VIO—within a long underground parking lot (as shown in Figure 7). In Area A (as shown in Figure 8), the vehicle first entered from the right-hand side, with the GNSS signal blocked throughout the entire route. The vehicle then traveled to the end of the parking lot (Area D) and returned after making a U-turn, as shown in Figure 9, and finally exited from the left-hand side of Area A. The red lines in Figure 7, Figure 8 and Figure 9 represent the performance of the reference system. The cyan lines denote the pure inertial result of the INS/GNSS framework, demonstrating a short trajectory in the along-track (north–south) direction and a slight shift in the cross-track (east–west) direction in Figure 9. The magenta lines illustrate the performance of the LIO system, which gradually drifted throughout the experiment. In Area D (Figure 9), the LIO system drifted along the heading angle, shifting the vehicle position in the east–west direction compared with the reference system. However, only a slight drift was observed in the along-track (north–south) direction. The green lines denote the performance of the VIO system, which exhibited a similar drift in heading angle to that of the LIO system but a considerably larger drift in the along-track direction in Area D (Figure 9).

**Figure 10:**
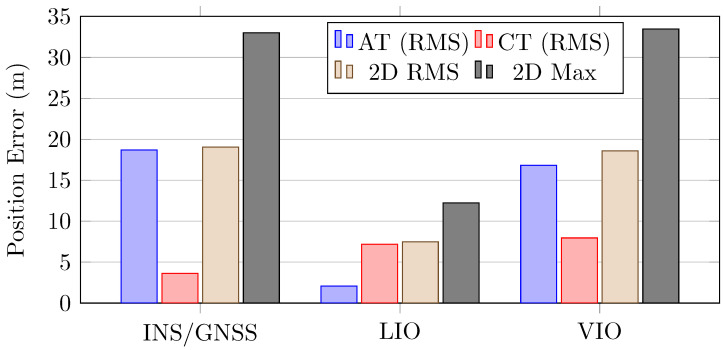
Position errors for the adopted INS/GNSS, LIO, and VIO systems.

Table 6 and Figure 10 present the position errors for the INS/GNSS, LIO, and VIO frameworks. The errors were projected onto the along-track (north–south), cross-track (east–west), and upward directions. The INS/GNSS framework and VIO system had smaller mean errors than the LIO system in the cross-track direction. However, the LIO system had the smallest mean error in the along-track direction and the highest two-dimensional position accuracy (Figure 9). The LIO trajectory aligned with the reference trajectory in the along-track direction.

**Figure 11:**
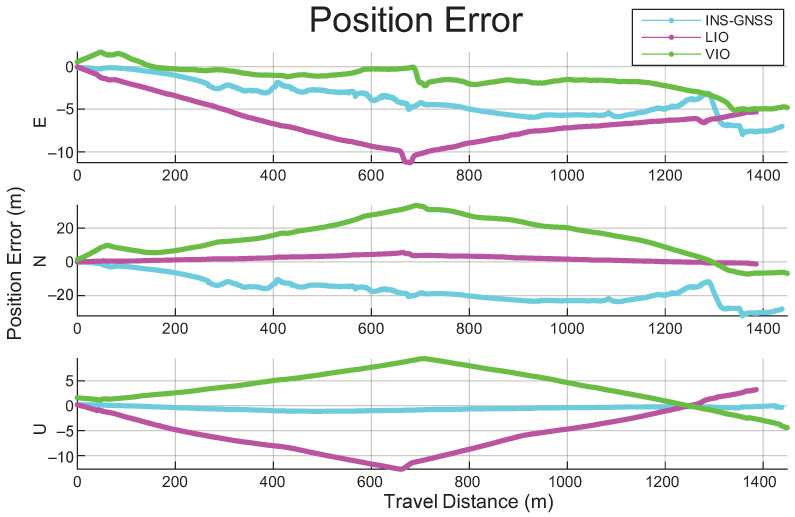
Position errors for the adopted INS/GNSS, LIO, and VIO systems over the entire travel distance.

Figure 11 shows the position error over travel distance for the INS/GNSS, LIO, and VIO systems. As expected, the INS/GNSS position error increased with travel distance. In contrast, the LIO and VIO systems showed a nearly symmetrical pattern; this may have been caused by interior orientation errors between the IMU and the odometry platform, even though all IMUs were aligned using the proposed calibration method.

### 4.3. Performance Evaluation—Multi-Sensor Fusion

**Figure 12:**
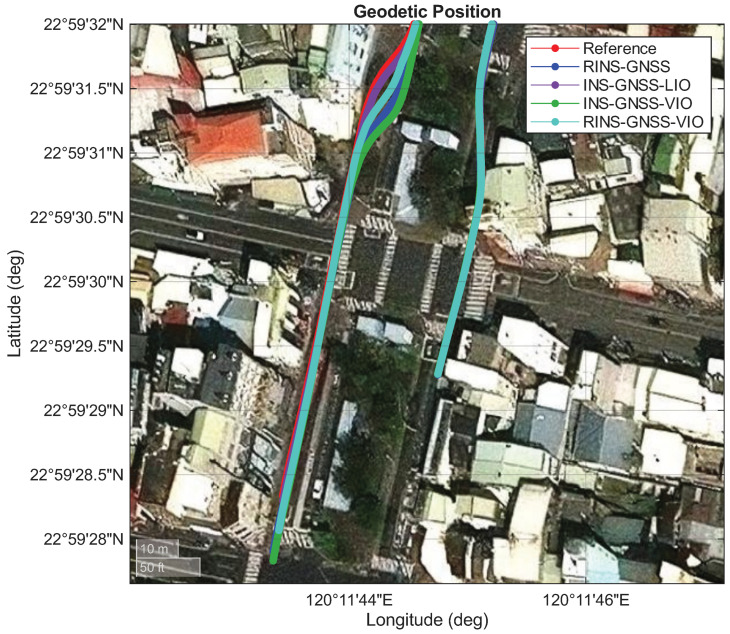
Performances of the adopted RINS/GNSS, INS/GNSS/LIO, INS/GNSS/VIO, and RINS/GNSS/VIO frameworks in Area A.

**Figure 13:**
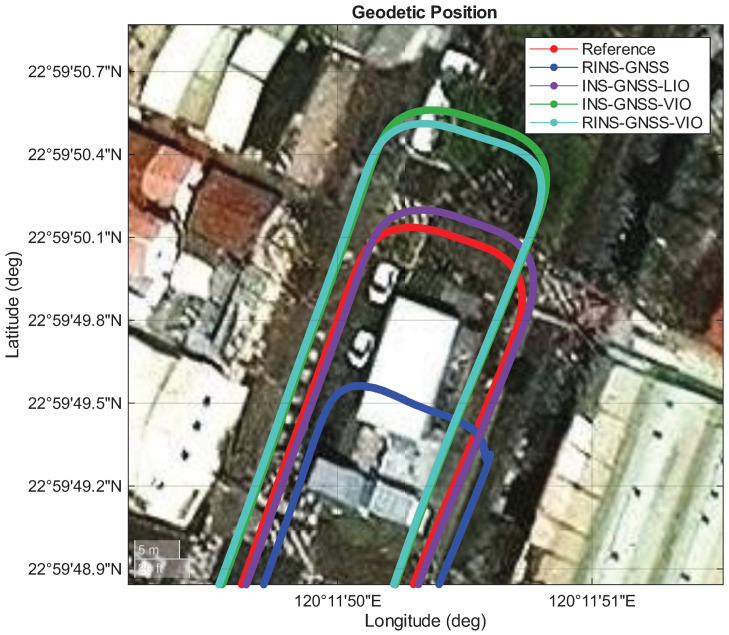
Performances of the adopted RINS/GNSS, INS/GNSS/LIO, INS/GNSS/VIO, and RINS/GNSS/VIO frameworks in Area D.

We compared the experimental performance of four fusion systems within the selected long underground parking lot: INS/GNSS/LIO, INS/GNSS/VIO, RINS/GNSS, and RINS/GNSS/VIO. The red line in Figure 12 represents the performance of the reference system. The blue line in this figure denotes the performance of the RINS/GNSS system, which exhibited less drift in the along-track direction than the INS/GNSS system. The purple line illustrates the performance of the INS/GNSS/LIO framework, which combined the best cross-track performance of the INS/GNSS system with the best along-track performance of the LIO system. The INS/GNSS/LIO framework showed the lowest 2D position error among the four fusion frameworks. The green line denotes the performance of the INS/GNSS/VIO framework, which exhibited drift both along the heading angle and in the along-track direction (Figure 13). Overshoot was also noted at the end of the underground parking lot (Figure 12). Finally, the turquoise line denotes the performance of the RINS/GNSS/VIO framework, which demonstrated better performance than the INS/GNSS/VIO and RINS/GNSS frameworks in the along-track direction.

**Figure 14:**
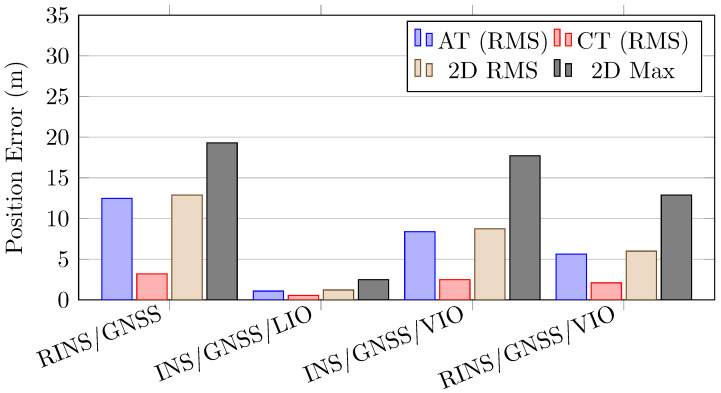
Position errors for the adopted RINS/GNSS, INS/GNSS/LIO, INS/GNSS/VIO, and RINS/GNSS/VIO frameworks.

Table 7 and Figure 14 presents the position error comparison between the RINS/GNSS, INS/GNSS/LIO, INS/GNSS/VIO, and RINS/GNSS/VIO frameworks. Compared with the single-IMU-based INS/GNSS framework, the RINS/GNSS framework demonstrated improvements in all statistical indices. The integration of the INS/GNSS framework into the INS/GNSS/LIO and INS/GNSS/VIO systems provided performance benefits in the cross-track direction. The INS/GNSS/LIO framework achieved a root mean square (RMS) 2D position error of 1.22 m, which meets the “Which Lane” accuracy level (1.5 m) requirement for lane-level accuracy applications, even in a GNSS-denied environment with GNSS outage for over 420 s without any pre-built map or prior environmental knowledge. After we identified the large performance gap between the INS/GNSS/LIO and INS/GNSS/VIO frameworks, we integrated the RIMU/GNSS framework with VIO to investigate whether this combination would achieve similar performance to that of the INS/GNSS/LIO framework. The result shows that the RINS/GNSS/VIO framework achieved an RMS 2D position error of 4.71 m, which was still far from that of the INS/GNSS/LIO framework.

**Figure 15:**
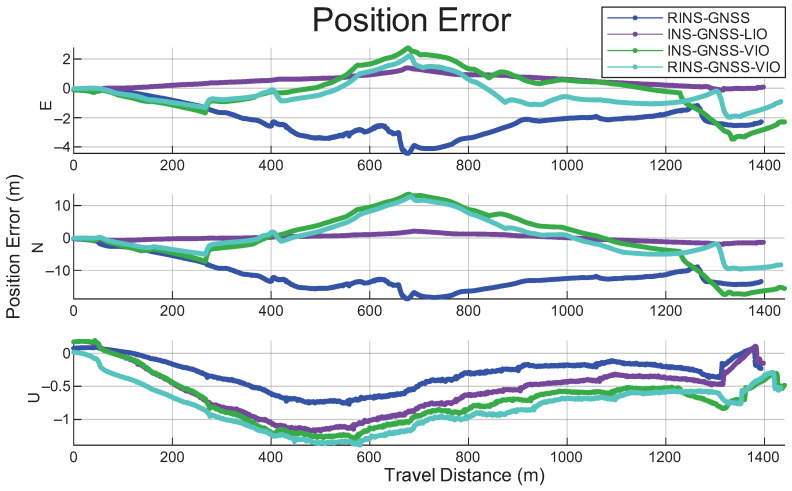
Position errors for the adopted RINS/GNSS, INS/GNSS/LIO, INS/GNSS/VIO, and RINS/GNSS/VIO frameworks over the entire travel distance.

Figure 15 shows the position error over travel distance for the RINS/GNSS, INS/GNSS/LIO, INS/GNSS/VIO, and RINS/GNSS/VIO systems.

**Figure 16:**
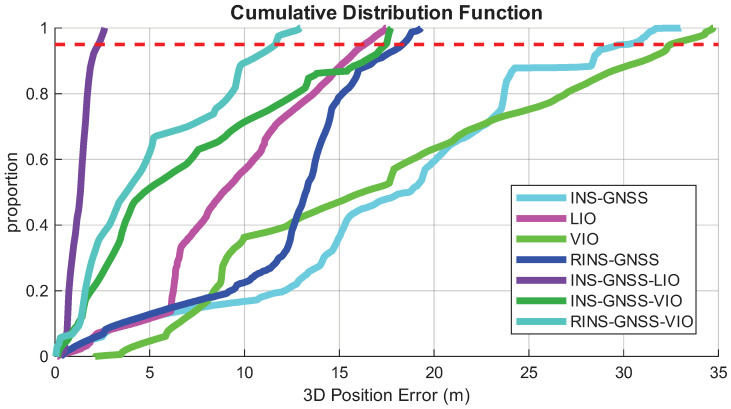
CDFs of the 3D position errors for the adopted INS/GNSS, LIO, VIO, RINS/GNSS, INS/GNSS/LIO, INS/GNSS/VIO, and RINS/GNSS/VIO frameworks. The red dashed line represents the 95% position error.

Figure 16 shows the 3D position error CDFs for all seven frameworks. The INS/GNSS framework exhibited a trend similar to that of the VIO system, with both having 95% position errors within approximately 33 m. The LIO system demonstrated the best performance, with the 95% position error remaining within 17 m. For the INS/GNSS framework, whether integrated with RIMU, LIO, or VIO, the 95% position errors were all within 18 m. The INS/GNSS/LIO framework achieved the best performance, with the 95% position error within 2.5 m. Despite the high performance of LIO, installing a vehicle with LiDAR is more expensive than equipping an RIMU or a camera.

A cost–performance analysis was also performed as part of this study, as summarized in Table 8. We gathered the costs of the sensors used in this study and compared their performance in our experiment. It should be noted that we chose an industrial camera for the PTP time-syncing function, which is more expensive than a consumer-grade camera. Furthermore, the reported cost of the LiDAR is the approximate cost of the Livox HAP LiDAR module, which was estimated according to our purchase price.

## 5. Conclusions

We systematically evaluated the performance of INS/GNSS, RIMU, LIO, and VIO navigation frameworks and developed a system calibration methodology using IMUs and external sensors as a baseline for comparison. LIO and VIO are limited by interior orientation errors associated with the used equipment. We calibrated all relations between sensors and the vehicle using an IMU such that any misalignment between the IMU and the LiDAR device (LIO) or camera (VIO) would produce an error in the navigation results. LIO system errors typically result from misalignment between the IMU and the LiDAR system. Such errors only accumulate in the cross-track direction, with adequate performance preserved in the along-track direction. Meanwhile, VIO system errors can result from misalignment between the IMU and the camera, as well as scale uncertainty. Even with IMU assistance, scale uncertainty and bias estimation remain highly correlated, as reflected in the observed VIO positioning errors in both the along-track and cross-track directions. Compared with LIO and VIO systems, an INS/GNSS framework calibrated using the proposed method, after INS error model well converged and motion constraints are applied, showed better positioning performance in the cross-track direction. Fusing LIO and VIO with an INS/GNSS framework can considerably improve positioning accuracy. In particular, the INS/GNSS framework compensates for the large position error of LIO in the cross-track direction, improving the RMS 2D position error of the LIO system from 7.48 to 1.22 m, thus ensuring that “which lane” accuracy (1.5 m) is achieved for land vehicle applications without any pre-built map or prior environmental knowledge, even in a GNSS-denied environment. Integration of the INS/GNSS framework with VIO also improved the RMS 2D position error of a VIO system from 18.59 to 8.74 m; however, the along-track RMS error (8.38 m) remained greater than the cross-track RMS error (2.48 m). Although incorporating an RIMU directly improves the measurement capability compared with an IMU, an RINS does not improve positioning accuracy to the same degree as LIO or VIO integration with an INS through mutual compensation. Notably, the implementation of an RINS is primarily beneficial for reducing system cost. In this study, we focused on cost-effective strategies involving MEMS-based IMUs and low-cost sensors, demonstrating that considerable improvements in the performance of sensors with different error characteristics can be achieved through appropriate compensation. For example, the INS/GNSS/VIO framework—which suffers from high along-track errors—can be further improved by incorporating an additional along-track velocity constraint, such as measurements obtained with an inexpensive wheel speed sensor or a radar sensor that measures absolute velocity.

## Figures and Tables

**Table 1:** Comparison of gyro bias instability and cost.

IMU	EPSON G320	EPSON G370	ASM330	ASM330 ×3
Gyro Bias Instability	<3.5°/h	<0.8°/h	2.9°/h *	1.6°/h
Cost (USD)	$2500	$10,000	$16	$48

* In our experience, the gyro bias instability of ASM330LHH could differ from 2 to 9°/h under different conditions.

**Table 2:** Specifications of the iNAV-RQH IMU.

iNAV-RQH	Accelerometer	Gyroscope
Bias Instability	<15 µg	<0.002°/h
Random Walk Noise	8 µg/$\sqrt{\mathrm{Hz}}$	$0.0018{}^{\circ}/\sqrt{h}$

**Table 3:** Specifications of the camera and lens.

Camera: Basler acA1300-75gc	
Resolution	1280×1024 pixels
Focal length	8 mm
Frame Rate	30 fps in VINS
Sensor Type	CMOS
Interface	Ethernet
Lens	ICL-DM0824I-5M
FOV	206.3×128.13 mm
Total Cost	USD 600

**Table 4:** Livox HAP LiDAR specifications.

LiDAR: Livox HAP Solid State LiDAR	
Maximum Measurement Range	150 m
Range Accuracy	<2 cm
Field of View (Vertical)	25°
Field of View (Horizontal)	120°
Angular Resolution (Vertical)	0.23°
Angular Resolution (Horizontal)	0.18°
Scan Points per Second	452,000 points/s

**Table 5:** Statistical results obtained in system calibration simulation (100 runs, boresight angle range of −30° to 30°).

Angle Error	Mean	STD	Max
Roll	−0.0074°	0.0006°	0.0087°
Pitch	0.0007°	0.0008°	0.0027°
Yaw	−0.0368°	0.0150°	0.0692°

Overall angle RMS error: 0.0404°.

**Table 6:** Position errors for the adopted INS-GNSS, LIO, and VIO systems.

	INS/GNSS				LIO				VIO			
Position Error (m)	AT	CT	U	2D	AT	CT	U	2D	AT	CT	U	2D
Mean	16.86	1.87	0.56	17.30	1.06	6.65	5.89	6.96	14.84	6.15	4.50	16.40
Max	32.98	−20.22	−1.14	32.99	12.24	11.11	−12.74	12.24	33.36	32.55	9.44	33.45
STD	17.59	3.58	0.37	7.98	1.81	7.14	4.41	2.74	16.65	7.81	3.63	8.77
RMS	18.70	3.62	0.65	19.05	2.08	7.18	7.00	7.48	16.81	7.96	5.22	18.59

**Table 7:** Position errors for the adopted RINS/GNSS, INS/GNSS/LIO, INS/GNSS/VIO, and RINS/GNSS/VIO frameworks.

	RINS/GNSS				INS/GNSS/LIO				INS/GNSS/VIO				RINS/GNSS/VIO			
Position Error (m)	AT	CT	U	2D	AT	CT	U	2D	AT	CT	U	2D	AT	CT	U	2D
Mean	11.62	1.74	0.34	12.03	0.91	0.47	0.61	1.08	6.46	1.32	0.73	6.80	4.34	1.16	0.82	4.71
Max	18.66	−19.18	−0.77	19.30	−2.47	2.22	−1.18	2.48	17.70	13.28	−1.29	17.71	12.88	11.65	−1.39	12.88
STD	12.28	3.09	0.24	4.61	1.07	0.55	0.37	0.55	8.13	2.48	0.37	5.49	5.44	2.09	0.34	3.70
RMS	12.48	3.19	0.40	12.88	1.08	0.55	0.70	1.22	8.38	2.48	0.81	8.74	5.62	2.09	0.89	5.99

**Table 8:** Cost–performance analysis of proposed fusion schemes used in our experiment.

Fusion Scheme	Sensor Configuration	Total Cost	2D RMS Error (m)	95% Error (m)
INS/GNSS	Single IMU ($16)	$16	19.05	∼30
RINS/GNSS	RIMU: 3 × IMU ($48)	$48	12.88	∼18
INS/GNSS/VIO	IMU ($16) + Camera ($600)	$616	8.74	∼18
RINS/GNSS/VIO	RIMU ($48) + Camera ($600)	$648	5.99	∼12
INS/GNSS/LIO	IMU ($16) + LiDAR * ($800)	$816	1.22	∼2.5

* The estimated cost of the Livox HAP LiDAR module, according to our purchase price. The price found on the official website is $1599. Note: All costs are in USD. The GNSS receiver cost is not included as it is common to all schemes.

## Data Availability

The data presented in this study are available on request from the corresponding author.

## Abbreviations

The following abbreviations are used in this manuscript:

CDF	Cumulative distribution function
EKF	Extended Kalman filter
GNSS	Global navigation satellite system
IMU	Inertial measurement unit
INS	Inertial navigation system
LIO	LiDAR inertial odometry
MEMS	Micro-electromechanical system
NHC	Non-holonomic constraint
RIMU	Redundant inertial measurement unit
RINS	RIMU inertial navigation system
VIO	Visual inertial odometry
ZIHR	Zero integrated heading rate
ZUPT	Zero-velocity update
